# Supplementary material for: Judging the unseen: The impact of onset controllability in shaping perceptions of defendants with traumatic brain injury
Source: PLoS One. 2025 Dec 8;20(12):e0323637. doi: 10.1371/journal.pone.0323637 (PMC12685182; doi:10.1371/journal.pone.0323637)
Supplement: S1 Table — (DOCX) [file pone.0323637.s001.docx]

**S1 Table. Comparative summary and synthesis of findings from onset controllability research in legal contexts**

| Study | Context | Sample | Onset Controllability | Verdict / Sentence Related Recommendations | Perceived Risk and Dangerousness | Empathy/Sympathy | Behavioural Perceptions |
| --- | --- | --- | --- | --- | --- | --- | --- |
| Present | UK. Case scenario based. Assault charge. No verdict or sentence presented at outset | General population (n = 173, 60.35% female, mean age = 34.85 years) | Directly examined: Onset Controllable (TBI), Onset Uncontrollable (TBI), and No-TBI Control | No significant main effect of onset controllability on severity of sentence or sentence recommendation ratings | No significant main effect – ratings not significantly different across conditions | More empathy / sympathy for defendant with TBI, irrespective of its onset controllability | Behavioural tendencies rated less negatively when TBI was present, irrespective of its onset controllability |
| Doyon et al. (2000) | US. Case scenario based. Homicide vs. Arson charge. Insanity verdict – found NGRI or guilty despite NGRI plea | Students (n = 196, 64.80% female, mean age = 21.7 years) | Indirectly examined: Onset Controllable (alcoholism or depression) vs. Onset Uncontrollable (Schizophrenia or PTSD) | Significant interaction between Onset Controllability and verdict - guilty verdict more appropriate for onset controllable; NGRI more appropriate for onset uncontrollable | Not examined | Not directly examined - less pity in response to onset controllable disorders | Not examined |
| Heath et al. (2001) | US. Case scenario based. Assault charge. No verdict or sentence presented at outset | Students (n = 220, 55% female, mean age = 21.77 years) | Indirectly examined: Excuses differing in ‘self-inflictedness’ level - High (cocaine dependency), Moderate (parental abuse syndrome), and Low (PTSD) | Significant main effect of self-inflictedness level - High condition more likely to receive a guilty verdict, a marginally longer sentence, be rated as more guilty, and perceived as presenting a less credible excuse | Not examined | Not examined (sympathy for victim, not defendant, was examined) | Not examined |
| Higgins et al. (2007) | US. Case scenario based.  Assault charge. No verdict or sentence presented at outset | Students (n = 96, 84.4% female, mean age = 22.58 years) and older adults (N = 96, 61.5%, mean age = 73.62 years) | Indirectly examined: Excuses differing in self-inflictedness level – High (cocaine dependency) vs. Low (Post-Traumatic Stress Disorder) | Significant main effect of self-inflictedness level –High condition rated as more guilty, and more likely to receive a guilty verdict and longer sentence | Not examined | More sympathy for defendant in Low vs. High self-inflictedness condition | Not examined |

*Note:* NGRI – Not guilty by reason of insanity; TBI = Traumatic brain injury; PTSD = Post-Traumatic Stress Disorder; Across the studies, conditions (i.e., onset controllable vs. onset uncontrollable; high vs. low self-inflictedness level) were presented as an excuse / mitigating factor.
