# Supplementary material for: Judging the unseen: The impact of onset controllability in shaping perceptions of defendants with traumatic brain injury
Source: PLoS One. 2025 Dec 8;20(12):e0323637. doi: 10.1371/journal.pone.0323637 (PMC12685182; doi:10.1371/journal.pone.0323637)
Supplement: Attachment — Submitted filename: Response to Reviewers.docx [file pone.0323637.s003.docx]

**Editorial and Reviewer Comments**

Thank you for your feedback. We have responded to each of your suggestions and queries in the list below, with our responses recorded in blue text. Please note that page and line numbers in our response correspond to the *unmarked* version of our manuscript.

(1) Adding a clear and concise diagram to justify your findings compared to other researchers will enhance clarity. Highlight key differences and similarities, ensuring the diagram is easily understandable and visually appealing. This will provide a better visual context for your results.

Thank you for your feedback. In response, we have produced a comparative summary and synthesis table (S1 Table) that summarises key differences and similarities between our study and past research on onset controllability (referenced on lines 621-622 and 707 in the discussion). We have opted for this format instead of a Venn diagram or flow chart, for example, for several reasons. Whilst our study has been informed by prior work on onset controllability, it represents the first time that onset controllability has been explored within the context of TBI. Therefore, the context, design, and dependent measures vary significantly across the present and prior work in the broader area. This variability across studies makes it challenging to create a single, visually appealing diagram that accurately represents the nuances of each without either oversimplifying the information, or risking a complex figure that does not bring additional clarity for readers. Instead, an accessible comparative table has allowed us to present the relevant details more succinctly and clearly, supporting readers to easily compare the different elements of each study (e.g., the manipulations, contexts, sample, methodology) as well as the findings (differences and similarities) across each. As such, it has also allowed us to add further detail to complement both the interpretation of study findings and our corresponding discussion of these findings, and the description and overview of each study cited within the manuscript body.

In addition to the above, we have also taken opportunity to correct a spelling error on line 178 and make a small adjustment to our description of a prior study in the introduction section (line 164).

**Journal Requirements**

(1) Please ensure that your manuscript meets PLOS ONE's style requirements, including those for file naming.

Levels heading and titles (sentence case) have been adjusted accordingly throughout the manuscript body. Additionally, the title page has also been updated (i.e., affiliations by number; formatting of corresponding author details; title in sentence case).

(2) When completing the data availability statement of the submission form, you indicated that you will make your data available on acceptance. We strongly recommend all authors decide on a data sharing plan before acceptance, as the process can be lengthy and hold up publication timelines. Please note that, though access restrictions are acceptable now, your entire data will need to be made freely accessible if your manuscript is accepted for publication. This policy applies to all data except where public deposition would breach compliance with the protocol approved by your research ethics board. If you are unable to adhere to our open data policy, please kindly revise your statement to explain your reasoning and we will seek the editor's input on an exemption. Please be assured that, once you have provided your new statement, the assessment of your exemption will not hold up the peer review process.

The data file is available on the OSF and is already available. Our data availability statement has been updated accordingly to reflect this.

<https://osf.io/ydc4p/?view_only=48523d0569e54d07928271b6669b5c4b>

(3) Please review your reference list to ensure that it is complete and correct. If you have cited papers that have been retracted, please include the rationale for doing so in the manuscript text, or remove these references and replace them with relevant current references. Any changes to the reference list should be mentioned in the rebuttal letter that accompanies your revised manuscript. If you need to cite a retracted article, indicate the article’s retracted status in the References list and also include a citation and full reference for the retraction notice.

The reference list has been reviewed for completeness. In response, two additional references [55, 56] have been added to the reference list (lines 990-994) that were previously omitted. Apologies for this oversight. Additionally, no papers have been cited that have retraction notices.
