# Supplementary material for: Judging the unseen: The impact of onset controllability in shaping perceptions of defendants with traumatic brain injury
Source: PLoS One. 2025 Dec 8;20(12):e0323637. doi: 10.1371/journal.pone.0323637 (PMC12685182; doi:10.1371/journal.pone.0323637)
Supplement: Attachment — Submitted filename: Response to Reviewers 22.07.2025.docx [file pone.0323637.s004.docx]

**Editorial and Reviewer Comments**

Thank you for your feedback. We have responded to each of your suggestions and queries in the list below, with our responses recorded in blue text. Please note that page and line numbers in our response correspond to the *unmarked* version of our manuscript.

Reviewer #1:

(1) In the results section, the ages (range) was described however was not added to the demographics table, this is a key factor which could influence the course of the outcome.

Participant ages ranged from 18 to 74 years (M = 34.86; SD = 13.64), as reported in the participants section of the manuscript (lines 230-231). To avoid unnecessary repetition, and because Table 1 focusses on categorical variables reported as Ns and percentages, we have not added age to demographic characteristics table.

(2) 2. The study, in both the methods or the results makes no mention of the occupation of the participants even though it mentions they are of general population in the supporting information. It's prudent these information are placed in the main write up especially in these two sections if possible and also describe or make mention of it in the abstract. If data was collected on the occupation of the participants they could be categorized and presented in the demographics in the results section.

Thank you for this comment. We did not collect data on participants' specific occupations (e.g., job titles or employment sectors). However, we collected and report a range of demographic characteristics in Table 1 (line 236), including gender, ethnicity, highest educational qualification, and employment status (e.g., full-time, part-time, unemployed, student, retired). These categories provide a broad overview of participants' socio-economic and employment contexts. The range of employment statuses and educational qualifications represents diversity expected from a general population sample. For example, unlike some prior research in this area, our participants were not limited to university students or a single educational group. In line with your suggestion, we have also amended the abstract to clarify that the sample was drawn from the general population (lines 19-20).

(3) In all the manuscript by Williams and Ledovskyte presents interesting findings on the perception TBI associated criminality in the UK. Generally the paper is well written and well presented.

Thank you for your helpful suggestions and positive comments.

Reviewer #2:

(1) This is a very relevant, interesting, and methodologically robust piece of research.

Thank you for your positive comments.

(2) My only comment concerns the explanations offered in support of the results of the study under Discussion. The authors propose various possible explanations for the fact that perceived controllability of TBI did not significantly affect participants sentencing related recommendations. However, they do not touch upon the very controllability factor in the hypothetical scenario. Possible explanations could also lie in the perceived controllability and moral dimension of the defendants behaviour prior to the accident, which are much less pronounced in the current study compared to the previous ones. Perhaps the respondents assessed the defendant’s failing to wear a protective equipment prior to the accident as (morally) less reproachable compared to e.g. long-term drug and/or alcohol abuse. Similarly, they might have assessed TBI in both scenarios (onset controllable and onset uncontrollable) as a consequence of an unfortunate one-time coincidence (and thus, in both cases as less controllable), in contrast to a repeating substance abuse behaviour in other studies.
    This hypothesis would also explain the study’s further finding that “in contrast to both theory and prior research suggesting that onset controllable conditions evoke less empathy, there was also no significant difference in empathy/sympathy ratings between the onset controllable and uncontrollable conditions”. Should the authors find this proposition relevant, they might briefly comment on it in discussion.

Thank you for this insightful suggestion. In response, we have revised our Discussion section (lines 630-642) to incorporate the possibility that participants may have perceived the behaviour leading to the injury in our onset controllable condition (i.e., failing to wear protective equipment) as relatively minor and less morally reprehensible than the behaviours used in prior studies (e.g., driving while intoxicated; cocaine dependency). We acknowledge that this difference in the moral salience and perceived culpability of the antecedent behaviour may have reduced the downstream impact of the onset controllability manipulation in our study, such as sentencing and empathy-related judgements.

(3) In lines 258-259 there is a small typing error: “Care was taken was avoid providing any specific motivations...”

This has been corrected accordingly, to “Care was taken to avoid providing any specific motivations….” (lines 256-257).
